# Performance evaluation of a novel fully-automated molecular diagnostics system Molecision R8

**DOI:** 10.1371/journal.pone.0349674

**Published:** 2026-05-19

**Authors:** Yanfang Mo, Xiaowen Wu, Chao Chen, Lixia Liang, Jianyin Su, Chengde Li, Yueying Wang, Ting Wang, Pu Sun, Zhonggang Fang, Zhifang Lin

**Affiliations:** 1 Department of Clinical Laboratory, The First People’s Hospital of Zhaoqing, Zhaoqing, China; 2 Research and Development Department, Shenzhen New Industries Biomedical Engineering Co., Ltd, Shenzhen, China; Children’s National Hospital, George Washington University, UNITED STATES OF AMERICA

## Abstract

**Objective:**

To evaluate the functionality of key modules of a novel fully-automated molecular diagnostics system Molecision R8 and the performance of the integrated system.

**Methods:**

The nucleic acid extraction and PCR detection modules were evaluated using HBV DNA assay through precision and comparison with Molecision MP-32 and HongShi SLAN-96, respectively. Instrument comparison of R8 with an open system was conducted using Molecision HBV DNA and *Chlamydia trachomatis*/*Ureaplasma urealyticum*/*Neisseria gonorrhoeae* (CT/UU/NG) triplex assays. Systemic comparison of R8 and combined reagents with Sansure system were conducted on HBV DNA, CT, UU, and NG assays of their own.

**Results:**

In modular evaluation, the imprecision of both modules was all below 5% and Passing-Bablok (PB) regression and Bland-Altman (BA) analysis showed closeness to y = x and biases below 0.15 lg IU/mL. Instrument comparison obtained a regression equation of y = 0.982x + 0.084 and a bias of 0.05 lg IU/mL for HBV DNA detection in serum and agreement rates all above 96.5% for CT, UU and NG detection in genital tract samples. In the systemic comparison, the regression equation was y = 1.024x + 0.096 and the bias was 0 lg IU/mL for HBV DNA. For CT, UU and NG, the agreement rates were also all larger than 96.5%.

**Conclusions:**

Analytical performance of Molecision R8 was superior or comparable to commercial nucleic acid purification and PCR systems. Molecision R8 alone or combined with related reagents are robust in measuring serum and genital tract samples. Overall, Molecision R8 holds strong promise for clinical use.

## Introduction

The COVID-19 pandemic has sounded a global alarm about the severity of infectious diseases, especially in the context of climate change which was shown to accelerate the transmission of infectious diseases [1]. Infectious diseases are illnesses due to invasion and colonization of human body by pathogenic microorganisms [2]. In addition to COVID-19 which temporarily ranked the first cause of global death in 2021, lower respiratory infections have remained in the top 5 leading causes since 1990 [3]. Accurate and rapid detection of specific microorganisms is of great importance not only to the healthcare of infectious diseases but to the containment of their community spread [4,5]. Furthermore, it could facilitate the epidemiological study of infectious diseases [6], thereby improving societal response to potential outbreaks [7]. The traditional methods for microorganism detection include culture and staining [8]. However, combining high accuracy with superior time- and labor- efficiency to the traditional microbiological methods, nucleic acid amplification testing (NAAT) is establishing its role as the gold standard for diagnosing infectious diseases [8–10].

NAAT is a type of molecular diagnostics technology for detecting a specific sequence of DNA or RNA, making it well suited to early identification of microorganisms [11]. NAAT is typically implemented with polymerase chain reaction (PCR) in clinical laboratories, which is highly susceptible to contamination from aerosol, specimens and amplicon [12,13]. To prevent contamination, clinical PCR laboratory is generally configured as adjoining rooms with physical separation reserved for supply storage, reagent preparation, nucleic acid extraction and PCR detection, respectively [14,15]. In addition, conventional PCR workflow requires stringent, intensive, and demanding manual operations [16]. Such complex laboratory layout and workflow inherently restrict the sample turnaround and testing throughput [17,18], which not only brings clinical laboratory more human resources costs but also limits the accessibility of PCR services to patients.

To address the aforementioned problems of laboratory PCR testing, fully-automated molecular diagnostics system was proposed as a solution [19]. Until now, there have been only a limited number of such platforms available on the market [20–22], which restricted the development of molecular diagnostics industry. Recently, an innovative fully-automated molecular diagnostics system Molecision R8 developed by Snibe Diagnostics (Shenzhen, China) has been launched. To facilitate the clinical application of this novel platform, the study aims to verify the performance of nucleic acid extraction and PCR detection modules of Molecision R8, and to validate its multiplex testing capability and its compatibility with diverse sample types.

## Materials and methods

### Sample collection

From October 2024 to May 2025, a total of 374 residual serum samples ordered for testing Hepatitis B virus DNA (HBV DNA) and another 365 residual genital tract samples for testing *Chlamydia trachomatis* (CT), *Ureaplasma urealyticum* (UU) or *Neisseria gonorrhoeae* (NG) DNA were collected at The First People’s Hospital of Zhaoqing. The serum samples of HBV DNA were used in the evaluation of the nucleic acid extraction and PCR detection modules, instrument comparison (the instrument of Molecision R8 compared with that of Snibe open system), and systemic comparison (combined system of Molecision R8 and related assays compared with Sansure detection system). The Sansure detection system (Sansure Biotech Inc., Changsha, China) was routine PCR detection system used in the hospital. The genital tract samples of CT, UU or NG were used for instrument comparison and systemic comparison.

The research was approved by the ethics committee of The First People’s Hospital of Zhaoqing (Approval No. B2024-05–02). The research adhered to the Helsinki Declaration and Good Clinical Practice (GCP) principles. All subjects signed an informed consent form for donating their residual biospecimens for future medical research.

### Modular evaluation of Molecision R8

**Fig 1:**
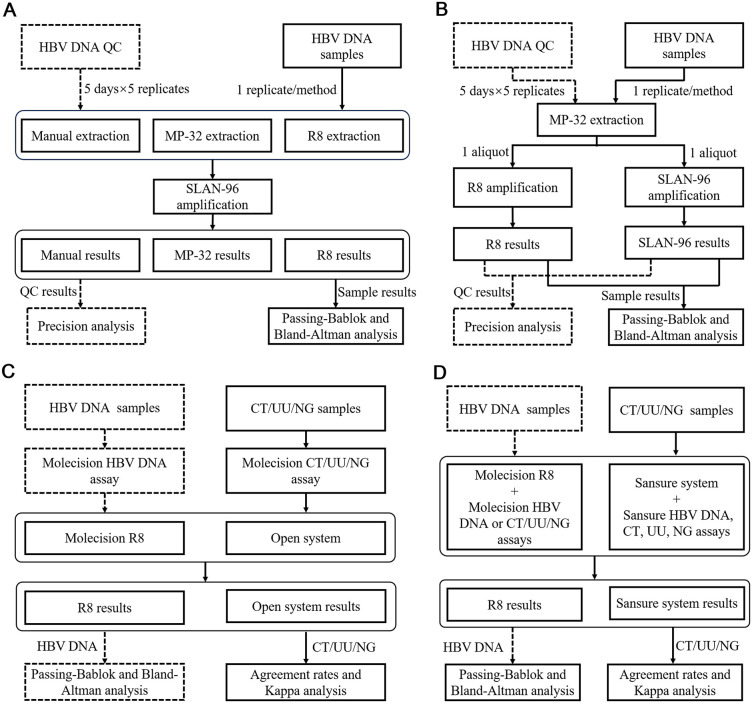
Experimental workflow for A) nucleic acid extraction module evaluation, B) PCR amplification module evaluation, C) instrument comparison, and D) systemic comparison.

The workflow for evaluating the nucleic acid extraction module of Molecision R8 was presented in Fig 1A. The same HBV DNA samples were divided and extracted either manually, using a Chinese FDA approved fully-automated nucleic acid purification system Molecision MP-32 (Snibe Diagnostics, Shenzhen, China), or the nucleic acid extraction module in tandem. Then, the processed samples were loaded to the same real-time PCR system SLAN-96 (HongShi, Shanghai, China) for HBV DNA detection. The same reagent kit was used in the manual method, MP-32 and R8 extraction module. The same extraction procedure with MP-32 and R8 was adopted by the manual extraction. To evaluate the precision, quality controls (QCs) were tested in 5 days with 5 replicates in each day using the same procedure.

As shown in Fig 1B, the PCR detection module was evaluated by extracting HBV DNA samples using MP-32. Following extraction, the samples were aliquoted and loaded to SLAN-96 and the PCR detection module of Molecision R8 for HBV DNA measurement, respectively. QCs were also tested to obtain precision estimates following the 5 days × 5 replicates protocol and the same assay procedure for evaluating the PCR detection module.

### Instrument and systemic evaluation of Molecision R8

Fig 1C and 1D summarized the workflow for instrument and systemic evaluation, respectively. In the instrument and systemic evaluation, method comparison studies were conducted with the open system consisting of MP-32 and SLAN-96 or the Sansure detection system as the reference methods. For instrument comparison, the same Molecision HBV DNA assay or Molecision CT/UU/NG triplex assay were used with both Molecision R8 and the open system. As to the systemic comparison, different HBV DNA, CT, UU, and NG assays were used in combination with their corresponding detection systems. Limit of detection (LoD) verification of Molecision CT/UU/NG triplex assay was conducted simultaneously on Molecision R8 and the open system as per Clinical and Laboratory Standards Institute EP17-A2. The samples with CT, UU, NG concentrations of LoD were prepared by diluting samples quantitated by digital PCR to LoD (400 copies/mL). Inconsistent samples in systemic comparison between R8 and Sansure were tested using Daan system (Daan Gene Co., Ltd. Guangzhou, China) for confirmation.

### Detection assays

Molecision HBV DNA assay is a quantitative assay that measures the concentration of HBV DNA in serum or plasma samples. Exogenous internal control (IC) system is adopted to monitor the nucleic acid extraction and PCR amplification process, with a target gene sequence of zucchini hydroxypyruvate reductase and its HEX-labelled probe. To prevent contamination, uracil-DNA glycosylase (UDG) was used to selectively hydrolyze the N-glycosidic bond of uracil in double-stranded or single-stranded DNA containing deoxyuridine (dU). The limit of detection (LOD) and limit of quantification (LoQ) are 2 IU/mL and 10 IU/mL, respectively. The results between LoD and LoQ will be reported as concentrations for reference only. The results below the LoD will be reported as <2 IU/mL if the IC shows a Ct ≤ 40, whereas an error will be reported if the IC shows a CT > 40.

Molecision CT/UU/NG triplex assay is a qualitative assay that detects the presence of CT/UU/NG in genital tract samples (vaginal swab and urethral swab). The IC system for the triplex assay consists of a target β-globulin gene sequence in the samples and its HEX-labelled probe. UDG is also incorporated to prevent contamination. The LoDs for CT, UU and NG are all 400 copies/mL with Ct < 40, whereas the positive cutoff value is Ct = 40. Therefore, a sample will be reported as negative if its Ct value is less than 40. The Sansure HBV DNA, CT, UU and NG assays were performed by the hospital staff following the respective instruction manuals.

### LoD sample preparation

Clinical samples tested positive for CT, UU or NG were serially diluted at volume ratios of 1:10, 1:10^2^, 1:10^3^, 1:10^4^ and 1:10^5^ and then measured by digital PCR to obtain absolute copies/mL for each dilution. The concentrations of the original samples were determined as the average of the concentrations back-calculated from the 5 dilutions. The LoD samples for CT, UU and NG were then prepared by serial dilutions of the original samples.

### Statistical analysis

Spearman correlation coefficient was used to analyze the correlation between methods. For quantitative HBV DNA assay, consistency was analyzed using Passing-Bablok (PB) regression and Bland-Altman (BA) plots. For qualitative CT, UU, and NG assays, consistency rate and kappa value were used for evaluating the agreement between methods.

## Results

### Evaluation of nucleic acid extraction module

Table 1 summarized the precision calculation using one-way analysis of variance (ANOVA) for HBV DNA results corresponding to manual extraction, MP-32 and R8 nucleic acid extraction module, respectively. The within-lab coefficients of variation (CVs) for the manual, MP-32 and R8 extraction were 7.91%, 2.52% and 2.08% for the high-level QC, respectively. For the low-level QC, the within-lab CVs were 24.78%, 6.63% and 4.67% for the manual, MP-32 and R8 extraction, respectively.

**Table 1:** Precision of HBV DNA assay for 3 extraction methods.

Extraction method	Mean conc. (lg IU/mL)	Within-run CV%	Between-day CV%	Within-lab CV%
Manual	2.50	15.17	19.60	24.78
	4.50	7.08	3.53	7.91
MP-32	2.50	2.05	6.31	6.63
	4.50	0.89	2.36	2.52
R8	2.50	2.55	3.91	4.67
	4.50	1.15	1.74	2.08

**Fig 2:**
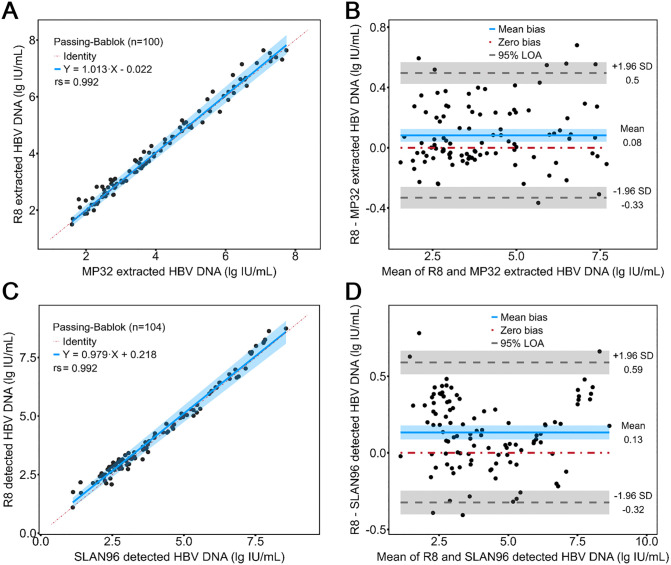
Agreement of nucleic acid extraction module and PCR detection module with MP-32 and SLAN-96. Passing-Bablok regression and Bland-Altman analysis for nucleic acid extraction module **(A, B)** and PCR detection module **(C, D)**.

Fig 2A and 2B depicted the PB regression and BA plot for comparing nucleic acid extraction module of Molecision R8 to Molecision MP-32. PB regression obtained a linear equation of y = 1.013x − 0.022 with a 95% confidence interval (CI) of (0.985, 1.044) for the slope and (−0.130, 0.091) for the intercept. In BA analysis, a bias of 0.08 lg IU/mL was observed and the percentage of samples within 95% limit of agreement (LOA) was 93%.

### Evaluation of PCR detection module

The precision of HBV DNA assay for either SLAN-96 or PCR detection module of Molecision R8 was shown in Table 2. The within-lab CVs of SLAN-96 detection were 2.42% and 0.43% for the low-level and high-level QCs, respectively. For the PCR detection module of Molecision R8, these within-lab CVs were 1.63% and 0.53%, respectively.

**Table 2:** Precision of HBV DNA assay for 2 detection methods.

Detection method	Mean conc. (lg IU/mL)	Within-run CV%	Between-day CV%	Within-lab CV%
SLAN-96	2.50	2.03	1.32	2.42
	4.50	0.38	0.20	0.43
R8	2.50	1.60	0.29	1.63
	4.50	0.46	0.27	0.53

In the comparison between the PCR detection module of Molecision R8 and SLAN-96 (Fig 2C and 2D), PB regression obtained a linear equation of y = 0.979x + 0.218 with a 95% CI of (0.940, 1.014) for the slope and (0.044, 0.379) for the intercept. The BA analysis showed a bias of 0.13 lg IU/mL, with 95% of samples within the LOA.

### Instrument and systemic evaluation in serum samples

**Fig 3:**
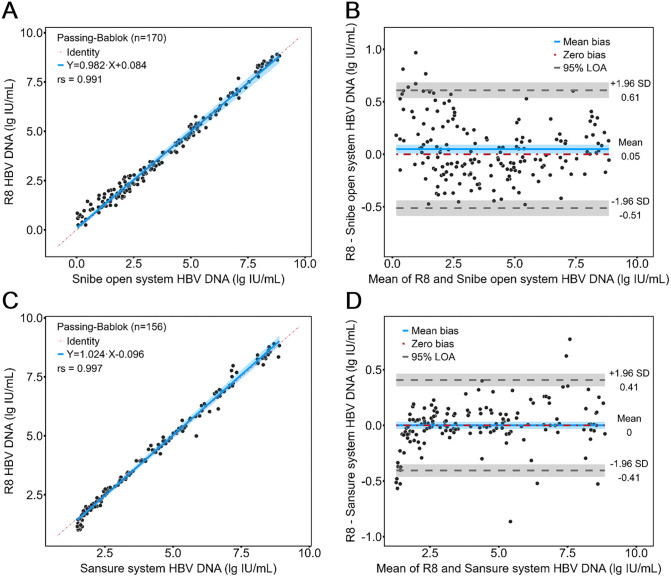
Agreement of Molecision R8 with the open system and Sansure system in HBV DNA testing. PB regression and BA plot for comparing R8 with **(A, B)** open system and **(C, D)** Sansure system.

To verify the robustness of Molecision R8 in measuring serum samples, R8 was compared head-to-head with the open system using HBV DNA assay, as illustrated in Fig 3A and 3B. PB regression showed a linear equation of y = 0.982x + 0.084, with the 95% CI for slope and intercept being (0.959, 1.003) and (−0.047, 0.190) respectively. There was an average bias of 0.05 lg IU/mL in BA analysis, with 97.06% of samples within the 95% LOA.

In addition, Molecision R8 was compared to a PCR testing system from Sansure. As indicated in Fig 3C, PB regression showed a linear equation of y = 1.024x + 0.096, with the 95% CI for slope and intercept being (1.008, 1.042) and (−0.157, −0.032) respectively. Fig 3D showed that the overall bias between R8 and Sansure system was 0 lg IU/mL and 94.23% of samples fell within the 95% LOA.

### Instrument and systemic evaluation in genital tract samples

Instrument comparison in measuring genital tract samples was conducted using qualitative CT, UU and NG assays. As summarized in Table 3, results of instrument evaluation were analyzed through agreement rate analysis. Three assays all showed high positive agreement rates, which were 100%, 100% and 98.15% for CT, UU and NG respectively. As the 3 assays were tested in the same batch of samples and NG had lower infection rate [23], the number of positive NG cases was only 54. With negative agreement rates of 98.89% for CT, 96.65% for UU and 99.04% for NG, the total agreement rates were determined to be 99.18%, 98.36% and 98.90% for CT, UU and NG, respectively. High kappa values of 0.98, 0.97 and 0.96 were also observed for CT, UU and NG. As summarized in S1 Table, Molecision R8 also had higher positive rates at the LoD levels for CT, UU and NG assays than the open system.

**Table 3:** Agreement rates for instrument comparison in CT/UU/NG testing.

Analyte	Class	Total N	Agreement N	Agreement rate (95% CI)	Kappa (95% CI)
CT	Positive	94	94	100.00% (96.07%−100.00%)	0.98 (0.95-1.00)
	Negative	271	268	98.89% (96.80%−99.62%)	
	Total	365	362	99.18% (97.61%−99.72%)	
UU	Positive	186	186	100.00% (97.98%−100.00%)	0.97 (0.94-0.99)
	Negative	179	173	96.65% (92.88%−98.45%)	
	Total	365	359	98.36% (96.46% ~ 99.24%)	
NG	Positive	54	53	98.15% (90.23%−99.67%)	0.96 (0.92-1.00)
	Negative	311	308	99.04% (97.20% ~ 99.67%)	
	Total	365	361	98.90% (97.22% ~ 99.57%)	

Systemic comparison in genital tract samples was also carried out using CT, UU and NG assays (Table 4). The positive and negative agreement rates were 96.74% and 99.30% for CT, 98.28% and 96.67% for UU, and 100% and 98.45% for NG, respectively. The total agreement rates were found to be 98.30%, 97.73% and 98.83% for CT, UU and NG, with kappa values of 0.96, 0.95 and 0.97 respectively. Confirmatory results of inconsistent samples tested by Daan system were summarized in S2 Table, with all results agreeing with Molecision R8.

**Table 4:** Agreement rates for systemic comparison in CT/UU/NG testing.

Analyte	Class	Total N	Agreement N	Agreement rate (95% CI)	Kappa (95% CI)
CT	Positive	92	89	96.74% (90.85%−98.88%)	0.96 (0.93-1.00)
	Negative	143	142	99.30% (96.15%−99.88%)	
	Total	235	231	98.30% (95.71%−99.34%)	
UU	Positive	174	171	98.28% (95.05%−99.41%)	0.95 (0.91-0.99)
	Negative	90	87	96.67% (90.65%−98.86%)	
	Total	264	258	97.73% (95.13%−98.95%)	
NG	Positive	42	42	100.00% (91.62%−100.00%)	0.97 (0.93-1.00)
	Negative	129	127	98.45% (94.52%−99.57%)	
	Total	171	169	98.83% (95.84%−99.68%)	

## Discussion

In this work, a novel fully-automated molecular diagnostics system Molecision R8 was evaluated in a bottom-up experimental design [24], with functional modules evaluated first, followed by the instrument alone and then the combination of instrument and reagents as a whole. The precision and bias of the nucleic acid extraction module of Molecision R8 were compared to a commercially available nucleic acid purification system Molecision MP-32. Molecision R8 showed the highest between-day and within-lab precision, followed by MP32 and manual extraction (Table 1), indicating that closed system of Molecision R8 could substantially reduce variation of nucleic acid extraction across batches, thereby improving within-lab precision. Although there were a number of studies evaluating diverse fully-automated molecular diagnostics systems [20,25,26], they seldom addressed the improvement of precision. Moreover, method comparison study exhibited a strong consistency between MP-32 and the extraction module, with a trivial bias of 0.08 lg IU/mL in BA analysis (Fig 2). Taken together, these results demonstrated decent functionality of the nucleic acid extraction module of Molecision R8.

The PCR detection module of Molecision R8 was also compared against a commercially available PCR system SLAN-96 on imprecision and bias. The within-lab imprecision was either smaller than or close to the open system (1.63% vs 2.42% for 2.5 lg IU/mL, 0.53% vs 0.43% for 4.5 lg IU/mL). The slope and the intercept of the PB regression were very close to 1 and 0, respectively. BA analysis also showed a relatively small bias of 0.13 lg IU/mL between the PCR detection module and the open system. These results were all evidence of the interchangeability between the PCR detection module and SLAN-96.

In the instrument and systemic evaluation of Molecision R8, we validated the performance of Molecision R8 in both serum samples and genital tract samples by comparing the instrument alone to the open system using the same reagent and by comparing Molecision R8 combined with bundled assays to the Sansure detection system. Molecision R8 demonstrated a substantially strong agreement with both the open system and the Sansure system in serum, as illustrated in Fig 3. However, in the comparison with the Sansure system, there was apparent bias at the lowest levels of HBV DNA near the limit of detection of the Sansure system. This is because the Molecision HBV DNA assay has a higher analytical sensitivity than the Sansure HBV DNA assay (2 IU/mL vs 30 IU/mL).

As to the validation in genital tract samples, positive, negative and total agreement rates for CT, UU and NG assays were all greater than 96% in the instrument and systemic comparisons, with all the kappa values larger than 0.95. In the instrument comparison, inconsistency lay primarily in the negative cases, which was due to higher sensitivity of Molecision CT/UU/NG assay on Molecision R8 than the open system as demonstrated by S1 Table. To resolve the discrepancy in the systemic comparison, Daan system was leveraged to test the inconsistent samples, whose results all agreed with Molecision R8. In this regard, the discrepancy between R8 and Sansure system could be due to difference in assay sensitivity, nucleic acid extraction efficiency and anti-contamination ability. In addition, difference in operators or laboratory environment could also be possible causes. Overall, these results all support the compatibility of Molecision R8 with genital tract samples. Furthermore, the consistency between the triplex Molecision CT/UU/NG assay and the singleplex Sansure CT, UU and NG assays indirectly uphold the multiplex capability of Molecision R8.

Overall, our results substantiated the strong potential of Molecision R8 for PCR testing in clinical settings. However, the study is limited by the single-center nature. In addition, carryover was not covered in this validation, even though pre-clinical evaluations have proved strong anti-carryover ability of Molecision R8 and no obvious carryover was observed in this study. Therefore, further research is needed to validate Molecision R8 in multi-center design with the inclusion of characteristics other than those included in this study. Furthermore, Molecision R8, as a new fully-automated molecular diagnostic system, could enhance the efficiency and accuracy of molecular testing and thus faster and more reliable clinical diagnosis and treatment decisions could be accomplished. Further studies should also be conducted on its performance in facilitating clinical diagnosis and treatment in comparison with traditional PCR lab workflows.

In conclusion, the study demonstrated superb analytical performance of Molecision R8 in comparison with regulatory body-approved nucleic acid purification and PCR systems. The nucleic acid extraction and PCR detection modules of Molecision R8 proved comparable or superior functionality to their approved counterparts. Systemic evaluation substantiated the reliability of Molecision R8 and related reagents in measuring serum and genital tract samples.

## Supporting information

S1 Table Limit of detection verification of Molecision CT/UU/NG triplex assay on R8 and open system. (DOCX)

S2 Table Confirmation of inconsistent samples in systemic comparison between R8 and Sansure by Daan system. (DOCX)
