# Supplementary material for: Performance evaluation of a novel fully-automated molecular diagnostics system Molecision R8
Source: PLoS One. 2026 May 19;21(5):e0349674. doi: 10.1371/journal.pone.0349674 (PMC13186364; doi:10.1371/journal.pone.0349674)
Supplement: S1 Table — (DOCX) [file pone.0349674.s001.docx]

Table S1. Limit of detection verification of Molecision CT/UU/NG triplex assay on R8 and open system.

| Analyte | N of replicates | N (%) detected by R8 | N (%) detected by open system |
| --- | --- | --- | --- |
| CT | 30 | 30 (100.00%) | 27 (90.00%) |
| UU | 30 | 30 (100.00%) | 29 (96.67%) |
| NG | 30 | 29 (96.67%) | 27 (90.00%) |
