# Supplementary material for: Performance evaluation of a novel fully-automated molecular diagnostics system Molecision R8
Source: PLoS One. 2026 May 19;21(5):e0349674. doi: 10.1371/journal.pone.0349674 (PMC13186364; doi:10.1371/journal.pone.0349674)
Supplement: S2 Table — (DOCX) [file pone.0349674.s002.docx]

Table S2. Confirmation of inconsistent samples in systemic comparison between R8 and Sansure by Daan system.

| Analyte | Sample | R8 | Sansure | Daan |
| --- | --- | --- | --- | --- |
| CT | 10.25-100 | - | + | - |
|  | 10.28-100 | - | + | - |
|  | 12.6-97 | - | + | - |
|  | 12.11-121 | + | - | + |
| UU | 11.13-123 | + | - | + |
|  | 11.19-98 | - | + | - |
|  | 12.11-121 | - | + | - |
|  | 215 | - | + | - |
|  | 232 | + | - | + |
|  | 235 | + | - | + |
| NG | 76 | + | - | + |
|  | 221 | + | - | + |
